# Editorial: Pain assessment and management in small animals

**DOI:** 10.3389/fvets.2023.1195275

**Published:** 2023-04-27

**Authors:** Caterina Di Bella, Alessandro Mirra, Petra Dmitrović

**Affiliations:** ^1^School of Bioscience and Veterinary Medicine, University of Camerino, Camerino, Italy; ^2^Department of Clinical Veterinary Medicine, Vetsuisse Faculty, University of Bern, Bern, Switzerland; ^3^Department of Clinical Sciences, Faculty of Veterinary Medicine, University of Zagreb, Zagreb, Croatia

**Keywords:** analgesic therapies, locoregional anesthesia, pain management, animal welfare, acute pain, chronic pain

Pain is a complex multidimensional experience, defined by the International Association for the Study of Pain (IASP) as “an unpleasant sensory and emotional experience associated with actual or potential tissue damage or described in terms of such damage” (1). It has been amply demonstrated that pain is a phenomenon shared by both humans and animals, and that it must be managed and treated with the same care and attention in both cases. Relieving pain is not only an ethical obligation, but it contributes to patient recovery and fosters a better veterinarian-owner relationship (2). However, despite advances in its recognition and treatment, it remains difficult to perform an early diagnosis and, consequently, to establish appropriate treatments. The inability of the animals to directly communicate with humans, and the tendency of some species to hide their painful state make this task even more complex (3). This leads to the continuous search for new techniques and methods for identifying pain, discriminating various painful conditions and, consequently, establishing a suitable therapy.

In this Special Issue, seven papers have been published which deal with different aspects of pain. Domínguez-Oliva et al. wrote a review on pain neurobiology in rodents and the possibility of detecting pain via the use of facial action units (FAU). Facial expressions have been shown to be a feasible non-invasive method to evaluate pain in acute, chronic, surgical, and neuropathic pain models, and to constitute a useful complementary tool for refining the use of rodents in research. Sylvain et al. tried to correlate intraoperative nociception with some respiratory parameters, hypothesizing that nociception may influence the rate of inspiratory gas flow (respiratory drive; Vt/T_i_) and the fraction of inspiratory time (T_i_/T_tot_) in anesthetized dogs. However, the large differences found among individuals suggest that these respiratory parameters may not be a proper target to correctly discriminate between the presence or absence of intraoperative nociception.

In the last decade, loco-regional anesthesia has allowed a breakthrough in the management of perioperative pain in veterinary species. To date, numerous techniques have been studied and applied in clinical practice. A great importance has been gained by interfascial blocks, aiming at depositing the local anesthetic in a fascial plane where neural targets are located. The TAP block (Transversus Abdominis Plane block) is one of these, and it is performed to provide analgesia of the abdominal wall, skin, subcutis and parietal peritoneum. Cavaco et al. evaluated the efficacy of this technique in dogs undergoing ovariectomy. In the study, 0.2 mL/kg of bupivacaine 0.25% was infiltrated bilaterally using both preiliac and retrocostal approaches. In agreement with previous literature, the TAP block allowed to reduce the signs of intraoperative nociception and to guarantee a lower analgesic demand in the post-operative period.

Despite the numerous loco-regional techniques developed in the last years, neuraxial anesthesia still remains one of the cornerstones when visceral analgesia is needed (e.g., peritonitis, pancreatitis, cholecystectomy). Sambugaro et al. performed a retrospective study comparing the intra- and postoperative efficacy of extradural versus systemic analgesia in dogs undergoing cholecystectomy. In agreement with what has been previously published, the requirement for intra and post-operative rescue analgesia was lower and the voluntary feed intake higher in patients receiving neuraxial anesthesia.

One of the main limitations described in the use of loco-regional blocks is their duration of action. In order to prolong the sensory but not the motor blockade, several drugs have been studied, either injected together with the local anesthetics or administered systemically. Stabile et al. compared the duration of a sciatic-femoral block performed with lidocaine, either associated with a constant rate infusion of dexmedetomidine or alone. The results obtained showed that the sensory block lasted longer in patients receiving dexmedetomidine, while no differences in recovery of patellar and tibial reflexes, proprioceptive function, and ability to walk were found among groups. The authors hypothesized that dexmedetomidine also inhibits delayed rectifier K+ and Na+ currents, reducing neuronal activity, and blocking the hyperpolarized activated cation current.

Unfortunately, incorrect analgesic treatment often leads to development of chronic pain, whose treatment is certainly more complex. Cannabinoids are widely studied for the management of chronic pain. Miranda-Cortés et al. conducted a review to summarize the pharmacokinetic and pharmacodynamic characteristics of cannabinoids, and highlighted their possible use in the treatment of acute, chronic and neuropathic pain, thanks to their ability in preventing both peripheral and central sensitization. Despite these promising results, non-steroidal anti-inflammatory drugs (NSAIDs) still remain the cornerstone of many pathological conditions, among which osteoarthritis (OA) in dogs. However, their use is often correlated with gastrointestinal ulceration, and it is contraindicated in the presence of renal insufficiency. Various combinations of nutraceuticals, as glucosamine hydrochloride and chondroitin sulfate, have also been described to improve OA signs, but the evidence of their effectiveness is variable. Kampa et al. have shown that dogs receiving PCSO-524 (compound of marine based fatty acids) and EAB-277 (combination of phospholipids extracted from krill oil together with lipid fractions of the green lipped mussel), but not those receiving glucosamine/chondroitin, resulted in significant improvements in peak vertical force within a few weeks, to a similar degree compared to carprofen.

In summary, the results of the aforementioned studies and reviews include new and interesting data related to the diagnosis and treatment of acute and chronic pain in veterinary species. The aspects covered and the results achieved show how a constant update in the field is needed and how new therapeutic targets could improve our current clinical practice.

## Author contributions

All authors listed have made a substantial, direct, and intellectual contribution to the work and approved it for publication.
